# Different roles of electromagnetic field experts when giving policy advice: an expert consultation

**DOI:** 10.1186/1476-069X-14-7

**Published:** 2015-01-21

**Authors:** Pita Spruijt, Anne B Knol, Arthur C Petersen, Erik Lebret

**Affiliations:** Institute for Risk Assessment Sciences, Utrecht University, Utrecht, The Netherlands; Centre for Sustainability, Environment and Health, National Institute for Public Health and the Environment (RIVM), Bilthoven, The Netherlands; Institute for Environmental Studies, VU University Amsterdam, Amsterdam, The Netherlands; Department of Science, Technology, Engineering and Public Policy, University College London, London, UK

**Keywords:** Roles of scientists, Electromagnetic fields, Uncertainty, Policy advice, Expert consultation, Q method

## Abstract

**Background:**

The overall evidence for adverse health effects of electromagnetic fields (EMF) at levels of exposure normally experienced by the public is generally considered weak. However, whether long-term health effects arise remains uncertain and scientific policy advice is therefore given against a background of uncertainty. Several theories exist about different roles that experts may take when they provide advice on complex issues such as EMF. To provide empirical evidence for these theories, we conducted an expert consultation with as main research question: What are the different roles of EMF experts when they provide policy advice?

**Methods:**

Q methodology was used to empirically test theoretical notions on the existence and determinants of different expert roles and to analyze which roles actually play out in the domain of EMF. Experts were selected based on a structured nominee process. In total 32 international EMF experts participated. Responses were analyzed using Principal Component Analysis and for the open questions we used Atlas.ti.

**Results:**

Four expert roles were found. Most striking differences between the four roles are whether experts consider current EMF policies adequate or not, whether additional –precautionary– measures are needed, and how experts view their position vis-à-vis policymakers and/or other stakeholders.

**Conclusion:**

This empirical study provides support for the so far mainly theoretical debate about the existence of different roles of experts when they give policy advice. The experts’ assessment of the degree of uncertainty of the issue turned out to be highly associated with their role. We argue that part of the controversy that exists in the debate regarding scientific policy advice on EMF is about different values and roles.

**Electronic supplementary material:**

The online version of this article (doi:10.1186/1476-069X-14-7) contains supplementary material, which is available to authorized users.

## Background

The public has been exposed to an increasing number of sources of electromagnetic fields (EMFs) for several decades. In addition to radio and television, these sources also include mobile phones (high-frequency fields) and electrical appliances in the home (low-frequency fields). The exposure associated with the quick proliferation of EMF sources, particularly from mobile phones, DECT phones and WiFi, has raised concerns about the possible adverse health effects.

The overall evidence for the adverse health effects of EMFs at levels of exposure normally experienced by the general public is considered weak [1–3]. Children are thought to be more sensitive to EMF exposure than adults, as their brains are still developing. Studies have shown an increased risk for childhood leukemia associated with low-frequency fields [4]. Thousands of studies have been performed on a wide array of health endpoints. The reviews on the association between EMF exposure and health effects in the general population show either no association or report insufficient and contradictory evidence [5–8]. The International Agency for Research on Cancer (IARC) classified EMFs as category 2B, meaning there is some evidence that EMFs may cause cancer in humans, but at present, the evidence is inconclusive [9, 10]. Due to the relatively recent worldwide rise of mobile phone use and rapid introductions of other new technologies, the long-term health effects remain uncertain, and concerns about such effects remain. Therefore, the current policy is given against a background of scientific uncertainty.

In addition to the IARC working group, other international assessments have been conducted to evaluate the potential carcinogenity from exposure to EMFs. The International Commission on Non-Ionizing Radiation Protection (ICNIRP) published information on the potential health risks from exposure to EMFs. The ICNIRP published guidelines for limiting exposure to EMFs (up to 300 GHz) in 1998 and reconfirmed the guidelines in a statement in 2009 [11], stating that no adverse health effects were expected when these guidelines were followed. The BioInitiative, a group of scientists and public health policy professionals, published an overview of what is known about the biological effects that occur when people were exposed to low-intensity EMFs [12]. The report concluded that “a reasonable suspicion of risk exists based on clear evidence of bioeffects at environmentally relevant levels, which, with prolonged exposures may reasonably be presumed to result in health impacts”. The BioInitiative experts proposed a precautionary approach, which was stricter than the ICNIRP guidelines. The BioInitiative report was not a systematic review, as opposed to the work of IARC and ICNIRP, and has therefore been criticized for the selective and incomplete use of the literature [13].

Several studies have shown the variation in expert advice and current national policies on EMFs [14, 15]. Some countries, such as Switzerland, Denmark and Australia, have adopted a precautionary approach on some EMF issues. Other countries have emphasized the absence of the proof of adverse health effects and have not implemented any policy interventions [16] beyond the existing ICNIRP guidelines.

In this study, we focused on the variance in expert advice. Given that experts usually have access to the same body of knowledge, the question arises how we can understand these differences in advice. When scientific data are inconclusive, experts have to advise in the face of uncertainty because scientific research is not able to provide a complete assessment of the risks or the effectiveness of policy measures. Such uncertainty provides room for a certain degree of subjectivity. Therefore, advice may be affected by normative ambiguity [17] such as personal opinions, values, worldviews and the larger social-cultural context, which could manifest in different attitudes and roles of experts, and subsequently, influence their policy advice.

Previously, we reviewed the theoretical work on the factors that may influence the way scientific experts advise policy makers on complex issues [18], such as EMFs. We found that such policy advice by experts can be investigated from a variety of perspectives, e.g., sociology, environmental studies, and political science. Therefore, the literature that we considered in our review has been published in a variety of journals, covering work from multiple scientific disciplines. The most important factors that were suggested as influencing the role of an expert when giving policy advice were the type of issue (level of uncertainty/complexity); the type of knowledge of the expert; the core values of the expert; the organization in which the expert works; the societal context (i.e., the position of science in society); and the ability of experts to learn and change their viewpoint. The review revealed that although well-elaborated theories exist (e.g., [19, 20]), there is limited empirical proof and underpinning.

We conducted an expert consultation using the issue of EMFs to provide more empirical evidence on expert roles and advice. Our goal was to empirically test theoretical notions on the existence of different expert roles and to analyze which roles actually play out in this domain, while exploring some of the factors that are associated with these roles. The following was the main research question: What are the different roles of EMF experts when they provide policy advice? The following sub question was also addressed: Which factors are associated with these different roles? We also explored the effects that different roles may have on policy advice.

## Methods

We selected and approached internationally renowned experts to explore the roles of experts when providing policy advice on EMFs and performed a Q survey to explore their viewpoints. The Q survey involved the formulation of statements (Q sample) about potential roles. Experts were asked to score and rank order these statements in a structured way. Finally, a Q-factor analysis was performed on the expert’s scores, and the different roles were interpreted. The sections below further describe the various steps.

### Nomination of participants and data collection

**Figure 1:**
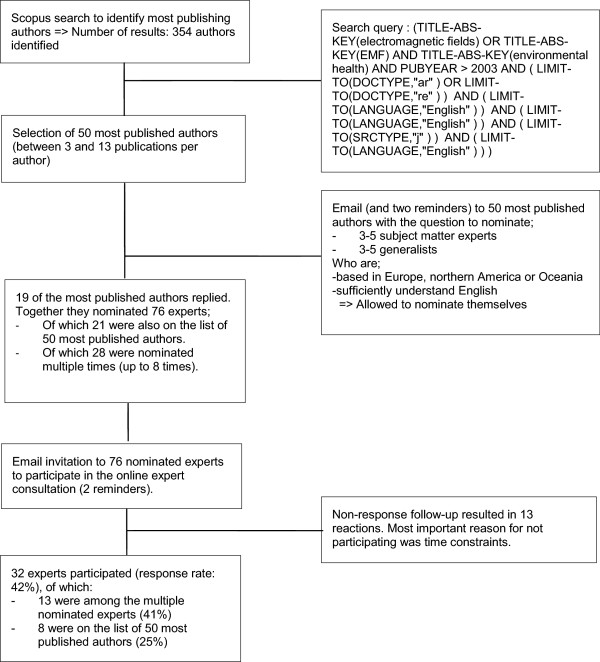
**Flow diagram outlining the expert nomination and selection process.**

We used a structured expert nominee process to obtain a list of prospective experts to take part in the Q survey [21]. Figure 1 shows an overview of the expert nomination and participation process. First, we used the digital search engine Scopus to identify the 50 most published experts (i.e., authors) on EMFs in relation to health issues. We limited the search to the period 2003–2013 to find experts who recently published on the topic. We assumed that these experts were up-to-date on the fields’ current scientific state of affairs. We emailed these 50 experts and asked them to nominate 3 to 5 subject matter experts and 3 to 5 generalists. Subject matter experts were fully involved in the scientific debate concerning EMFs and were seen as influential in the domain of EMFs. Generalists were familiar with the scientific debate concerning EMFs and were well-known for giving policy advice. All of the nominated experts were required to be based in Europe, Northern America or Oceania and had to sufficiently understand English. The experts were allowed to nominate themselves. Non-responding experts received two reminders by email. In total, 97 experts were nominated. The nominated experts were asked via email to participate in our online consultation. The online consultation was conducted using POETQ [22], which is a Partnership Online Evaluation Tool with Q methodology. Non-responding experts received two reminders by email. After these reminders, non-respondents received a follow-up email asking them to indicate the most important reason for not participating.

### Q methodology

Q methodology was used to explore the different expert roles in the field of EMFs. Q methodology was developed in the 1930s as a technique for studying human subjectivity [23]. This technique involves asking participants to sort a number of subjective statements based on their personal level of agreement/disagreement with the statements. The resulting Q sorts, which represent the viewpoints of individuals, are used to identify clusters of shared ways of thinking that exist among groups of people [24]. These clusters are identified statistically using factor analysis. An important assumption in Q methodology is that a limited number of distinct clusters exist for any particular issue [25]. An extensive description of the history, function and reliability of Q methodology can be found in previous studies [25–28].

### Q sample

The 38 Q statements (see Additional file 1) were compiled by the authors based on a pilot study [29], on our literature review [18] and on input provided by colleagues working in the domain of EMFs. They included different aspects of the expert roles and advice, including type of issue (level of uncertainty), organization in which the expert works and societal context (position of science in society). Three factors mentioned in the literature review were not incorporated in the Q sample: type of knowledge of the expert (participants were considered to be a relatively homogenous group); core values of the expert (implicitly incorporated in statements; explicit consideration would demand a separate Q sort); and the ability of experts to learn and change their viewpoint (in order to test this, several measurement points would be necessary). The statements were numbered randomly. The balance, clarity and simplicity of the set of statements and the proper functioning of the online data collection program (POETQ) were pre-tested with the help of three respondents who did not take part in the final study.

All of the participating experts rank ordered the 38 statements. First, each statement was categorized into one of three piles: agree, disagree and neutral. Consequently, all statements were rank ordered, pile by pile, over a forced quasi-normal distribution with scores representing the level of agreement, ranging from completely agree (+4) to completely disagree (−4).

### Statistical analysis

**Figure 2:**
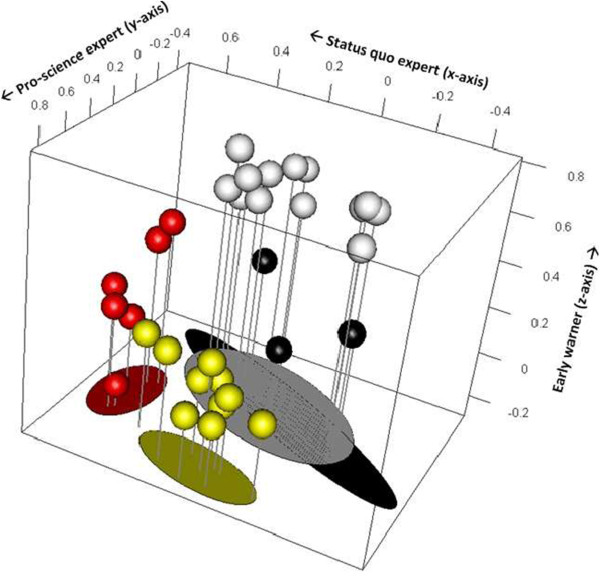
**Visualization of participants clustered in four roles: Early warners, 13 experts (white); pro-science, 10 experts (yellow); status quo, 6 experts (red); and issue advocates, 3 experts (black).** The X, Y and Z-axes show the different roles with their factor scores. Note that the axis for the issue advocate is not represented in the figure.

The PQmethod version 2.33 was used to analyze the correlation and factoring of the Q sorts. A Q sort consisted of the complete rank ordering of the statements as scored by one participant. Using Principal Component Analysis (PCA), a statistical correlation matrix was produced to summarize the similarities in views among participants. Next, clusters of similar viewpoints were identified. Part of the PCA was identifying the highest number of computed factors that hold at least three significantly loading Q sorts. We performed an analysis extracting three, four and five factors to find the most relevant number of factors. A Varimax rotation was applied to optimize the distance between factors. Subsequently, a characteristic Q sort distribution was calculated for each factor based on the standardized factor scores. This distribution revealed the statements that scored similarly within each cluster and therefore gave an idea of the common viewpoints represented by each factor. Next, we analyzed the overall consensus statements to gain an impression of the issues most EMF experts agree on, regardless of the factor they score significantly on. Then, we interpreted the differences between factors based on the so-called distinguishing statements. Given the three factors X, Y and Z, a distinguishing statement for factor X is a statement that received a score in factor X that is significantly different from the corresponding score in factors Y and Z. The authors then labeled each factor. The results of the PCA were visualized using statistical software package R (see Figure 2).

### Analysis of open questions; key scientific issue and policy advice

In addition to the Q-sort statements, we asked the experts two open questions. The first question was “What would you call the key scientific issue on EMF at this time?” The second question was “If you were asked to provide policy advice on EMF, what concrete policy measure would you recommend?” The answers were analyzed with the qualitative data analysis program Atlas.ti, version 6.2. This program was used to systematically analyze unstructured data, such as text. The program provides tools to give descriptive codes to primary data material, in this case, the written answers to the two open questions. The descriptive codes were used to structure the data and detect patterns in the respondents’ answers.

To detect whether a relationship could be traced between the roles of experts (i.e., the result of the PCA) and the content of their policy advice, we first broadly structured the experts’ answers (i.e., policy recommendations in answer to the second open question) in broad categories of policy measures. These categories were derived from three secondary sources: scientific literature, policy documents and conversations with experts. A total of 28 experts gave 34 distinctive policy recommendations. One expert could give several recommendations; thus, the total number of recommendations could exceed the number of respondents. Policy recommendations were analyzed both as one set, as well as distributed over the different factors.

### Additional questions

Finally, three additional questions were asked using Likert scales ranging from 1–5 as answer categories. These questions concerned other factors possibly associated with an expert’s role, but not yet included in the Q statements. These questions were (1) “I can give my advice independently and uncensored by my corporate hierarchy (independence)”; (2) “My research has had a direct influence on policy choices made (influence)”; and (3) “I think there is a high degree of uncertainty about the health risks posed by EMFs (level of uncertainty)”.

## Results

A total of 32 EMF experts participated in our consultation (see Figure 1). Table 1 shows the selected background variables of the participants, including demographic and employment details. In summary, the average age of the experts was 58 years, 41 percent were female, and 47 percent provided policy advice as their primary task. The majority of the experts were professor/researcher or director of research institutes. Common fields of expertise included epidemiology, public health, toxicology, risk assessment, biology and risk communication.

**Table 1:** **Background variables of the 32 participants**

***Demographics***		
Gender	Mean age	Nationality
	*(st. dev.)*	
59% male	58 *(8.8)*	Italian (5); U.S. (5); French (3); German (3); Swedish (3); Dutch (3); Austrian (2); Swiss (2); Australian (2); British (1); Finnish (1); Greek (1); Hungarian (1)
***Employment characteristics***		
**Field of expertise**	**Type of position**	**Type of employer**
Public health; Epidemiology; Risk/exposure/radiation assessment; Policy; Biology (cell/statistics/medical); Toxicology; Risk communication	(Senior) Researcher (11); Professor (10); Head/Director/Manager (7); Advisor (4);	University (13); Research Institute (10); Government (4); NGO (3); Industry (2); Independent Advisory Body (1)

The statistical analysis revealed four factors, i.e., four different sets of distinct statement patterns, which we called roles. In this study, we defined a ‘role’ as a cluster of distinct viewpoints shared among a group of scientific experts. The results are summarized in Table 2. The four roles indicated in this table illustrate the differences between viewpoints of EMF experts and yield a total explained variance of 56 percent. All 32 experts (i.e., sorts) were considered for the interpretation of the roles. Based on the distinguishing statements and factor scores, we interpreted the four roles and subsequently labeled them as follows: (1) early warners; (2) pro-science experts; (3) status quo experts; and (4) issue advocates. Figure 2 shows a visualization of the experts clustered per role.

**Table 2:** **Summary of main characteristics of the four expert roles**

Role	Key characteristics	Statements most strongly agreed with (+3 and +4) and least strongly agreed with (−3 and −4) – see numbers and corresponding statements in Additional file 1	No. of respondents ***(expl. var.)***	Summary of typical advice (based on 2nd open question)
Early warners	Disagreement with current policies. Transparency about methods, assumptions and personal preferences. More research. Precautionary measures.	(+) 18 21 25 26 34 (−) 2 11 22 24 29	13 *(18%)*	Precautionary measures. Develop new more stringent policy standards.
Pro-science	Evidence-based policy. Monitor risks. Not humble about contribution of science to society.	(+) 13 14 15 29 32 (−) 12 23 24 28 35	10 *(17%)*	Evidence-based policy, ALARA and ICNIRP guidelines*
Status quo	Agreement with current policies. No need for additional regulatory measures. Evidence-based policy.	(+) 13 14 20 22 26 (−) 5 6 16 23 28	6 *(11%)*	Evidence-based policy, ALARA and ICNIRP guidelines*
Issue advocates	Interaction with policy makers and stakeholders. More sources than science. No need to explicate differences of opinion between experts.	(+) 2 9 10 14 26 (−) 4 11 12 15 37	3 *(10%)*	-*(advice from 1 expert)*

*The differences between status quo and pro-science experts included the following: humble attitude of scientists and value of citizens’ knowledge.

The following sections describe the four different roles, based on the distinguishing statements. There was one issue that most experts seemed to agree on, namely that when scientific knowledge is inconclusive, policymakers have the task of dealing with the resulting uncertainty (statement 13). Because most experts agreed, this statement does not distinguish between roles, as is shown by the similar factor scores (see Additional file 1).

### Role 1: early warner

The ‘early warners’ role was shared by 13 experts and explained 18 percent of the total variance. The early warner experts strongly agreed that the risks and uncertainties of EMFs warrant significant investment in additional research (statement 21). They also agreed that when research results were translated into policy advice, experts should be completely open about the methods they use and the assumptions they make (statement 26). According to the early warners, differences of opinion among experts should be made explicit (statement 34). In addition to being open and explicit about differences of opinion, experts should also be transparent about their personal preferences with regard to the policy alternatives and the motivation for these preferences (statement 25).

The early warners *dis*agreed with the current policies on EMFs (statement 29—this is in contrast to the other three roles). In addition to the need for more research and transparency in communication about research, the early warners stated that just monitoring the situation is not enough (statement 22) and that additional precautionary measures are needed to protect public health and the environment (statement 18 and 23). The early warners did not feel tempted to initiate stakeholder cooperation (statement 2), in contrast to the issue advocates.

### Role 2: pro-science expert

The ‘pro-science expert’ role was shared by 10 experts and explained 17 percent of the total variance. The pro-science experts strongly agreed that new policies should be based entirely on the best available scientific knowledge (statement 20). The focus should be on evidence-based policy (statement 5 and 17), and there is no need for scientists to be humble about the possible contribution of science in solving societal problems (statement 32). They felt that experts’ personal values should stay separate from their policy advice (statement 24). According to the pro-science experts, the risks and uncertainties of EMFs require monitoring, and there is no need for additional measures, such as precautionary/regulatory measures or significant investment in additional research (statements 21, 22, 23 and 29). The pro-science experts were convinced that their views on the risks of EMFs do not differ very much from those of their colleagues (disagreement with statement 28). Experts holding one of the three other roles also tended to disagree with statement 28, but less strongly.

### Role 3: status quo expert

The ‘status quo expert’ role was shared by 6 experts and explained 11 percent of the total variance. A characteristic of status quo experts was their neutral and satisfied assessment of the current situation regarding EMFs. They strongly agreed with current policies on EMFs (statement 29—note that early warners have an opposite score on this statement) and thought that legislation and regulation is the best way to manage the possible health problems concerning EMFs (statement 4). The status quo experts believed that the risks and uncertainties of EMFs require monitoring, but there is currently no need for additional regulatory measures (statement 22). Furthermore, according to the status quo experts, their role was to address specific questions posed by policymakers (statement 15), and when they advise, they try to keep their personal values separate from the policy advice (statement 24). The status quo experts *dis*agreed with the idea that they should actively approach politicians to present their points of view on EMFs (statement 35). Status quo experts also *dis*agreed with the statement that knowledge of the general public is of less value to policymakers than expert knowledge (statement 17) and gave a neutral score to the statement that new policies should be entirely based on the best available scientific knowledge (statement 20).

The correlation with role 2 was high (0.58). There were two notable divergent viewpoints. First, the status quo experts agreed on the viewpoint that scientists should be humble about the role of science in solving societal problems (statement 32), whereas the pro-science experts *dis*agreed. Second, the status quo experts *dis*agreed on the viewpoint that the knowledge of citizens was of less value to policy makers than expert knowledge (statement 17), whereas the pro-science experts agreed.

### Role 4: issue advocate^a^

The ‘issue advocate’ role was shared by 3 experts and explained 10 percent of the total variance.

A distinct characteristic of the issue advocates was their intensive interaction with policymakers and other stakeholders (statement 12 and 15). The issue advocates tried to use their scientific knowledge to actively direct policy (statement 10), and they were personally motivated to initiate stakeholder cooperation in their research on EMF (statement 2). The issue advocates viewed it as their task to recommend the policy option that they considered best (statement 9). However, they felt that scientific knowledge was not the only source of information to consider when new policies are created (statement 20). According to the issue advocates, it was not necessary to make differences of opinion among experts explicit when they gave policy advice. They also believed that striving for consensus among experts did not best serve policymakers (statement 34 and 37).

### Variables associated with the expert roles

All of the experts provided us with information on their perceived independence, perceived influence on policy, perceived key scientific issue regarding EMFs, geographical location and their assessment of the degree of uncertainty about the health risks of EMFs. The first statement, “I can give my advice independently and uncensored by my corporate hierarchy”, received an average score of 3.9 on a Likert scale from 1–5 (ranging from disagree to agree), and there were no noticeable differences between the expert roles. Information on whom experts gave advice to and whether advice was given as in individual or representing an employer did not result in noticeable differences between expert roles either. The second statement, “My research has had a direct influence on policy choices made,” received an average score of 3.7 without significant differences between the expert roles. The third statement, “I think there is a high degree of uncertainty about the health risks posed by EMFs”, received the lowest average score of 2.8, with a marked difference between the expert roles. Namely, the pro-science experts gave an average score of 1.9, and the early warners gave an average score of 3.5. Clearly, the degree of uncertainty about the health risks was perceived differently between these two groups. Geographical location seems to be another influencing factor. The early warners were based in the US, Europe and Australia, whereas the other three expert groups consisted predominantly of Europeans.

The analysis of the answers to the first open question, “What would you call the key scientific issue on EMF at this time?,” resulted in one key scientific issue in the field of EMFs: the health effects of exposure to EMFs. This issue was mentioned by 26 of the 32 experts. Some experts specified the possible health effects, e.g., electro hypersensitivity, neurodegenerative diseases, cancer and negative effects on well-being.

### Proposed policy advice and expert roles

The analysis of the answers to the second open question, “If you were asked to provide policy advice on EMFs, what concrete policy measure would you recommend?”, resulted in a rather clear differentiation of proposed policy advice distributed over the expert roles (see Table 2). The early warners focused on the necessity to develop new standards and implement precautionary measures, such as creating preventive policies for children and informing the public on ways to reduce their exposure. The status quo and pro-science experts both focused on evidence-based policy. They recommended adopting the ALARA principle and the ICNIRP guidelines. The three issue advocates did not propose enough policy measures in our questionnaire. Overall, several experts asked for more research and emphasized the need to communicate to the public about research results. From the Q-sort, we saw that pro-science experts did not agree that a significant investment in research was needed (statement 21), whereas early warners strongly agreed with this statement. Overall, the qualitative analysis of the proposed policy advice confirmed the results of the factor analysis and showed that there was a relationship between an expert’s role and the policy advice s/he proposed.

## Conclusions and discussion

We conducted an expert consultation using Q methodology on the issue of EMFs to test theoretical notions on the existence of different expert roles and to see what factors were associated with these roles. The main research question was: What are the different roles of EMF experts when they provide policy advice? The following sub question was also addressed: What influences these differences? In addition, we explored the effect that different roles had on possible policy advice. We found four distinct expert roles that were labeled as (1) early warners; (2) pro-science experts; (3) status quo experts; and (4) issue advocates. The early warners disagreed with the current EMF policies. They agreed that more research and precautionary measures were needed and stated the importance of the transparency about methods, assumptions and personal preferences. The pro-science experts agreed that evidence-based policy was legitimate and stated that scientists should not be humble about the contribution of science to society. They preferred to monitor the risks of EMFs. The status quo experts agreed with the current policies on EMFs and saw no need for additional regulatory measures. Finally, Issue advocates agreed that scientists should interact with policymakers and stakeholders. They stated that there was no need to explain the differences of opinion between experts. We found a high correlation (0.58) between roles two and three; the other correlations were 0.35 and lower. The most striking differences between the four roles were whether current policies were adequate or not, whether additional precautionary measures were needed, and how the experts viewed their position vis-à-vis policymakers and/or other stakeholders.

According to the literature [18], the most important factors that influenced the role of an expert when giving policy advice are the following: type of issue (level of uncertainty/complexity); type of knowledge of the expert; core values of the expert; organization in which the expert works; societal context (i.e., the position of science in society); and the ability of the experts to learn and change their viewpoints. A comparison of the results of the literature review with the results of the Likert scale questions and, specifically, statements 18, 21 and 23 of our Q sort showed that the level of uncertainty and the context (i.e., geographical location), seemed to be associated with an expert’s role. The reported level of uncertainty differentiated highly between the EMF experts. The early warner experts perceived a much higher level of uncertainty than the pro-science experts. There was variation between the roles on the agreement about the best measures (e.g., more research, precautionary measures). The most notable difference was between the early warners and the pro-science experts. The first group of experts said there was a high level of uncertainty and believed that the risks and uncertainties of EMFs warrant significant investment in research, as well as precautionary measures. The second group of experts reported that there was a low level of uncertainty and believed the contrary viewpoint about more research and precaution. Apparently, a different assessment and interpretation of the level of uncertainty by an expert was associated with their expert role when providing policy advice. Regarding the context, we only asked respondents about their geographical location and at what type of institute they work. More in-depth questions about context (laws, policies in a specific country, party experts work for etc.) would be interesting research questions for a follow-up study. Another point taken from the literature was the suggestion to improve the way in which experts advise on complex issues by democratizing science, e.g., through public participation and stakeholder dialogues [30–35]. In our consultation, we found little support for broadening the advice process. In fact, only the issue advocates seemed to be willing to actively involve the public and other stakeholders in the advisory process (see scores on statements 2, 6, 11 and 17). Public participation is considered important, but our study showed that few experts actually engage in it. Another suggestion to improve the advice was a professional attitude of humility [36, 37]. The minimal support for statement 36 that scientists should ‘speak truth to power’ in their policy advice indicated some degree of humility (see also statement 32).

Furthermore, almost all of the non-Europeans were early warners. The two that were not advised the adoption of the ICNIRP guidelines. This may signify that the European experts in this study showed relatively less need for developing new standards and precaution than their American and Australian colleagues, although this distribution could be coincidental, due to the relatively small sample size. The sample of respondents was the result of a structured expert nomination and selection process. Additional selection criteria, such as the quality of journals that experts published in, could have been applied to the first step of the selection process. Because we were looking for scientists that also provide policy advice we chose to use group knowledge of the experts in order to select our participants instead of investing more time in selecting top scientists. The non-response follow-up research indicated that time constraints constituted the most important reason for not participating. This gave no particular indication of bias, although we cannot exclude the possibility of differences between respondents and non-respondents. One respondent was solely self-nominated. All other respondents were nominated by at least one colleague. An overrepresentation of worried scientists was possible, and this might have depended on funding opportunities. Worried scientists may be more prone to perform and publish research when there are scarce resources, rather than when a research field is well funded.

There is an ongoing debate among researchers about the best analysis strategy when using Q methodology. There are mainly two different ways that are described and advocated: principal component analysis (PCA) in combination with a varimax rotation and centroid analysis in combination with a manual rotation. Both strategies include arbitrary selection criteria, such as the minimum number of respondents loading significantly on a factor. We tested both strategies on our data in a sensitivity analysis. We found a large overlap between the two approaches. Both of the analyses showed three very similar factors, and the fourth factor in the PCA analysis seemed to be split into two separate factors in the centroid analysis. The centroid analysis yielded sorts from 25 experts for the interpretation, whereas the sorts of all 32 experts were included in the PCA analysis. Both of the analyses show substantial similarities in results. Therefore, we decided to use PCA, based on the argument that PCA enabled us to incorporate the highest number of experts in our results.

In the 1980s, Q methodology was mainly applied using face-to-face interviews. More recently, web-based approaches were developed that appeared to perform well [38]. We used the online consultation tool POETQ. Due to our international set of respondents, the geographical distances made it impossible to perform face-to-face interviews. This may have resulted in higher rates among respondents of non-responding, misinterpreting statements or other parts of the consultation and providing less elaborate answers to the open questions. On the other hand, the advantage of using an online tool was that we were able to include more respondents and respondents from geographically separated countries.

We compared our list of respondents to the membership lists of the ICNIRP and the BioInitiative participants. The results pointed towards a relationship between involvement with one of these groups and the attributed expert roles. It was interesting that experts who participated in our consultation thought that their views on the risks of EMFs did not tend to differ from those of colleagues (statement 28). However, the results of our research clearly indicated differences in roles and viewpoints.

Our study confirmed that different distinct roles and viewpoints existed within the community of EMF experts. This research also suggested that the indicated level of uncertainty was one of the factors associated with the EMF experts’ roles and, most likely, their policy advice. Further study is needed to determine if this was a causal relation and if this also applies to other environmental health issues. This empirical study provided support for the mainly theoretical debate about the role of experts when they give policy advice. These first empirical findings need corroboration from other empirical studies and on other issues. Additionally, we need to better understand both determinants of roles as well as its effect on policy advice and debate. Based on these results, we argue that part of the controversy that exists in the debate regarding scientific policy advice is about different values and roles (i.e., normative ambiguity [17]). These insights may lead to a better understanding of the processes and differences in the results of scientific policy advice on complex issues.

### Endnote

^a^We used a label earlier coined by Pielke Jr [19]. We saw many similarities between Pielke’s description of the Issue advocate and ours but noted that the connotation was not exactly the same.

## Electronic supplementary material

Additional file 1: **Statements with factor scores (i.e., factor Q-sort values).** (DOCX 21 KB)

## Abbreviations

ALARA: As low as reasonably achievable
DECT: Digital enhanced cordless telecommunications
EMF: Electromagnetic fields
ICNIRP: International commission on non-ionizing radiation protection
IARC: International agency for research on cancer
POETQ: Partnership online evaluation tool with Q methodology
PCA: Principal component analysis.
