# Supplementary material for: Different roles of electromagnetic field experts when giving policy advice: an expert consultation
Source: Environ Health. 2015 Jan 21;14:7. doi: 10.1186/1476-069X-14-7 (PMC4417251; doi:10.1186/1476-069X-14-7)
Supplement: Supplementary file 1 — Additional file 1: Statements with factor scores (i.e., factor Q-sort values). (DOCX 21 KB) [file 12940_2014_843_MOESM1_ESM.docx]

**Appendix: Statements with factor scores (i.e., factor Q-sort values)**

| No | Statement | Role 1:  Early warners | Role 2:  Pro-science | Role 3:  Status quo | Role 4:  Issue advocate |
| --- | --- | --- | --- | --- | --- |
| 1 | As an expert, I think I should cooperate with stakeholders in assessing the health risks of EMFs. | 1 | 0 | 1 | 1 |
| 2 | I feel personally motivated to initiate stakeholder cooperation in my research on EMFs. | -3 | -1 | -1 | 3 |
| 3 | I expect the government to coordinate the governance process on EMFs. | 0 | 2 | 2 | 0 |
| 4 | I think that possible health problems concerning EMFs are best managed by legislation and regulation. | 0 | -1 | 2 | -3 |
| 5 | In my opinion, public health and environmental problems related to EMFs are too complex for only evidence-based policy. | 0 | -3 | -2 | -1 |
| 6 | As an expert, I should take the perspectives of the general public into account in my research. | -2 | -3 | -1 | 1 |
| 7 | As an expert, it is my duty to maintain continuous dialogue with policymakers. | 0 | 0 | 0 | 0 |
| 8 | As an expert, it is my responsibility to inform policymakers about all possible policy options and their potential consequences. | 1 | 0 | 0 | 2 |
| 9 | As an expert, it is my task to recommend the policy option that I consider best. | 1 | -1 | -1 | 4 |
| 10 | I try to use my scientific knowledge to actively direct policy. | -1 | -1 | -2 | 4 |
| 11 | In my opinion, science should be limited to systematic knowledge production. | -3 | 1 | -1 | -3 |
| 12 | I think there should be strict separation between scientists who do research and policymakers who build policy on that research. | -2 | -1 | -3 | -4 |
| 13 | When scientific knowledge is inconclusive, I think policymakers have the task of dealing with the resulting uncertainty. | 2 | 3 | 3 | 2 |
| 14 | I think that scientific research should contribute to solving societal problems. | 2 | 3 | 4 | 3 |
| 15 | My only involvement in politics is to address specific questions posed by policymakers. | -1 | 0 | 3 | -4 |
| 16 | I think that public anxiety is a good motivation for policy action, even when there is no scientific explanation for the anxiety. | -2 | -3 | -2 | 0 |
| 17 | In my opinion, knowledge of the general public is of less value to policymakers than expert knowledge. | -1 | 2 | -2 | -1 |
| 18 | If the health and environmental impacts of a project involving EMFs were highly uncertain, I would advise precautionary measures to protect public health and the environment. | 3 | -1 | 1 | 0 |
| 19 | I expect future technological innovations to reduce the negative effects of EMFs on health and the environment. | -1 | 0 | -1 | -1 |
| 20 | I think new policies on EMFs should be based entirely on the best available scientific knowledge. | 2 | 4 | 0 | -2 |
| 21 | I believe the risks and uncertainties of EMFs warrant significant investment in additional research. | 4 | -2 | 0 | 0 |
| 22 | I believe the risks and uncertainties of EMFs require monitoring but there is currently no need for additional regulatory measures. | -4 | 4 | 2 | 0 |
| 23 | I believe the risks and uncertainties of EMFs warrant significant investment in precautionary measures | 2 | -4 | -3 | -2 |
| 24 | In addition to scientific knowledge, I preferably incorporate my personal values in my policy advice. | -4 | -2 | -4 | -1 |
| 25 | I think policy makers are best supported when experts are transparent about their personal preferences with regard to the policy alternatives and the motivation for these preferences. | 3 | 1 | 0 | 1 |
| 26 | In giving policy advice, I think experts should be completely open about the methods they use and assumptions they make. | 4 | 3 | 2 | 3 |
| 27 | I think I should inform policy makers about the science underlying my policy advice. | 1 | 2 | 1 | 1 |
| 28 | My views on the risks of EMFs tend to differ from those of my colleagues. | -2 | -4 | -3 | -1 |
| 29 | I agree with current policies on EMFs. | -3 | 1 | 4 | 0 |
| 30 | I am very interested in the political debate surrounding my research. | -1 | -1 | 1 | 2 |
| 31 | I think the primary task of a scientist is to publish in peer-reviewed scientific journals. | 0 | 1 | -1 | 0 |
| 32 | I think scientists should be humble about the role of science in solving societal problems. | 0 | -2 | 3 | 2 |
| 33 | I think that scientific output should be assessed by an extended peer community of all who are affected by the issue. | 0 | -2 | -1 | -2 |
| 34 | I think that differences of opinion among experts should be made explicit when giving policy advice. | 3 | 1 | 1 | -1 |
| 35 | Just as NGOs and industry do, I think scientists should actively approach politicians to present their points of view on EMFs. | 0 | 0 | -4 | -2 |
| 36 | I think scientists should ‘speak truth to power’ in their policy advice. | 1 | 2 | 0 | 1 |
| 37 | I think policy makers are best served when experts strive for consensus in their policy advice. | -1 | 0 | 0 | -3 |
| 38 | I primarily work in science because I like the intellectual challenge. | 1 | 1 | 1 | 1 |
